# Venomous Arachnid Diagnostic Assays, Lessons from Past Attempts

**DOI:** 10.3390/toxins10090365

**Published:** 2018-09-10

**Authors:** Camila Dias-Lopes, Ana Luiza Paiva, Clara Guerra-Duarte, Franck Molina, Liza Felicori

**Affiliations:** 1 Departamento de Bioquímica e Imunologia, UFMG, Belo Horizonte 31270901, Brazil; 2 Colégio Técnico (COLTEC), UFMG, Belo Horizonte 31270901, Brazil; 3 Fundação Ezequiel Dias (FUNED), Belo Horizonte 30510010, Brazil; analubpaiva@gmail.com (A.L.P.); claragd@gmail.com (C.G.-D.); 4 Sys2Diag UMR 9005 CNRS Alcediag, 34000 Montpellier, France; franck.molina@sys2diag.cnrs.fr

**Keywords:** arachnids, diagnosis, envenomation, spiders and scorpions, antivenom

## Abstract

Diagnostic tests for arachnid accidents remain unavailable for patients and clinicians. Together with snakes, these accidents are still a global medical concern, and are recognized as neglected tropical issues. Due to arachnid toxins’ fast mechanism of action, quick detection and quantification of venom is required to accelerate treatment decisions, rationalize therapy, and reduce costs and patient risks. This review aims to understand the current limitations for arachnid venom identification and quantification in biological samples. We benchmarked the already existing initiatives regarding test requirements (sample or biomarkers of choice), performances (time, detection limit, sensitivity and specificity) and their validation (on animal models or on samples from envenomed humans). Our analysis outlines unmet needs for improving diagnosis and consequently treatment of arachnid accidents. Hence, based on lessons from past attempts, we propose a road map for raising best practice guidelines, leading to recommendations for future progress in the development of arachnid diagnostic assays.

## 1. Introduction

The class Arachnida has over 60,000 described species. Most of them are not threatening for humans, but spiders and scorpions have a special apparatus for injecting venom associated with a venom gland that can strike human victims. They are the main thing responsible for accidents caused by arachnids, representing a worldwide health problem [1,2]. In terms of spiders, five major genera have been reported as being most relevant for human accidents (*Loxosceles*, *Latrodectus*, *Phoneutria*, *Atrax* and *Hadronyche*) [3,4,5,6,7]. In the case of scorpions, the majority of the one million cases reported annually [8] are caused by members of the Buthidae family (*Leiurus*, *Androctonus*, *Buthus*, *Tityus*, *Centruroides*, *Mesobuthus* and *Parabuthus*) [9]. 

The main therapeutic treatment for these accidents is based on antivenom administration [6,10,11]. However, the treatment of bitten/stung patients is not straightforward because of misdiagnosis or late diagnosis, mainly in the case of spider accidents. Typically, spider bites are not witnessed, the spider is not captured for identification, and symptoms are generally formulated with indirect evidence [5,12]. For instance, many black spiders in eastern Australia are often mistaken for the funnel-web spider and, therefore, patients bitten by a black spider, even in mild accidents, follow the protocol stated for funnel-webs, with the patient being discharged from hospital only after 4 h of observation [6].

Likewise, in 2013, there were more than 2400 patients of spider bites admitted to USA healthcare facilities; however, more than 95% of these cases presented none or minor medical outcomes, indicating the need for a diagnostic assay to differentiate relevant accidents from those that do not pose a threat to humans [13].

Furthermore, different studies have shown that many arachnid accidents are reported in non-endemic regions, where spiders of the reported genus have never been found by entomologists [14,15,16]. In addition, many conditions of different etiologies (such as infection by *Staphylococcus*) bring out skin injuries similar to lesions caused by spider bites, leading to wrong diagnosis by clinicians [12,17].

In many accidents with scorpions, clinical and biochemical laboratory analyses are insufficient to support the diagnosis [18,19]. In addition, although general symptoms occur soon after the sting, in some cases they can occasionally be delayed for several hours, which can complicate diagnosis [1]. Moreover, the improvement of scorpion envenoming management requires the establishment of a correlation between blood venom concentrations and envenoming grades (mild, moderate and severe) for antivenom immunotherapy optimization [20,21].

Identification and quantification of venoms in victims is crucial for effective patient treatment, to achieve antivenom effectiveness, and to improve epidemiological data. It is unfortunate that arachnid bite diagnostic assays remain unavailable. In fact, detection of arachnid venom in biological samples is not an easy task. The amount of venom injected by scorpions and spiders is very low (microgram or milligram range) and, hence, subject to variation. Regarding venom biodistribution, the concentration found in patient samples such as blood and urine can be very small or undetectable using current methods.

Since no arachnid diagnosis kit exists on the market, this review aims to learn from past experiences and to propose directions for future development.

## 2. In Vitro Assays for Identification and/or Quantification of Arachnid Venoms

### 2.1. Spiders 

Most studies on the identification and/or quantification of medically important arachnid venoms are dedicated to *Loxosceles* spiders (Table 1). Loxoscelism (the term used to define accidents caused by *Loxosceles* spiders) is the most common and clinically important accident resulting from spider bites. Patients who might not feel the bite or disregard minor symptoms frequently underestimate this kind of accident. Most signs and symptoms of cutaneous Loxoscelism (70% of cases), such as burning pain, edema, blister, ecchymosis, paleness and necrotic wounds take weeks to heal and appear only several hours after the bite [4]. The diagnosis is often made late, generally after 72 h, once skin lesions are well developed, and patients invariably receive multiple investigations before it is recognized [6]. In some cases, a corrective plastic surgery may be required to correct tissue injury and, in less frequent cases (13–16%), patients could develop systemic Loxoscelism (characterized by acute intravascular haemolytic anaemia) [6].

To diagnose *Loxosceles* envenomation in previous studies, researchers inspected skin exudate, skin biopsy and hair in or close to the lesion, once a large proportion of the venom has apparently concentrated in the region. The presence of venom in blood samples was also investigated, the favored sample used to identify/quantify venom from other spider species and from scorpions (Table 1 and Table 2). 

Interestingly, when skin exudate was analyzed, using saline-immersed swab from rabbits experimentally envenomed with *Loxosceles* venom, McGlasson and colleagues could detect venom up to 21 days after its injection. In this work, the average amount found corresponds to approximately 0.0001% of the venom initially injected, and it is claimed that the assay could detect in the range of picograms of venom [22]. Barret and co-workers also identified *Loxosceles* venom in 90% of guinea pig skin exudate assayed by passive hemagglutination inhibition assay [23]. In the case of human bitten patients, Stoecker and collaborators [24] identified *Loxosceles* venom three days after the bite, and Keklikci and co-workers [25] 13 days after lesion outbreak. The amount of venom found in human samples was higher than the one found in experimentally envenomed rabbits. This could be explained by the amount of venom injected into the victim (around 50 μg) compared to amounts experimentally injected in rabbits (only 5 μg of venom) [26].

Distinct groups have also worked with biopsy samples (from rabbits and patients), although it is considered an invasive procedure that causes great discomfort in patients. Despite this fact, this procedure could give an idea of the percentage of *Loxosceles* venom that remains in the bite/injection site. To obtain these samples, a 2–4 mm dermal biopsy is collected with a disposable biopsy punch from the necrotic area or an area close to the lesion. Although a work by McGlasson and colleagues did not find any detectable venom in biopsy samples from rabbits experimentally envenomated [22], another two works from Gomez and colleagues found a significant amount of *Loxosceles* venom in this type of sample [27,28]. These differences could be due to different administration routes used by the groups: McGlasson’s group used subcutaneous injection whereas Gomez’s group used intradermal injection. The quantity of venom recovered from this type of sample seems to be higher than from samples obtained from skin exudate, since approximately 0.1% of injected venom was detected one day after injection [27]. The amount of venom detected in biopsies appears to decrease within days after injection [28]. The biopsy of only one clinical case was inspected, and *Loxosceles* venom was detected in the nanogram range, four days after the bite [29].

Hairs extracted close to the bite site were also investigated in some studies. These hairs, with the follicular base included, were removed with sterile forceps from the adjacent area of the skin lesion or from the injection site. Venom could be measured in these samples up to 7 days after experimental injection in rabbits [28] and, in one clinical case, was detected 4 days post-envenomation [29]. The venom concentration estimated by the assays was in the order of pg/100 µL in experimental animals and ng/100 µL in the clinical case. Again, the higher concentration found in humans could be due to the higher amount of venom injected by the spider compared with experimental injection.

Even though serum is the most collected biological sample in a hospital environment, this type of specimen does not seem to be ideal for detection of *Loxosceles* venom. Immunoassays conducted with serum samples of experimentally envenomated rabbits [28] and a clinical case of Loxoscelism [24] failed to detect venom in this sample, even when the assay had a sensitivity on the order of picograms. Only Chávez-Olortegui and collaborators were able to detect *Loxosceles* venom in blood using experimentally envenomed mice [30]. It is worth mentioning that this animal model does not develop local skin lesions, which could concentrate venom in this organ, theoretically decreasing circulating venom. The group studied venom kinetics in experimentally envenomated mice, and venom could be detected from 0.5 h until 8 h after venom subcutaneous injection on the order of ng/mL [30]. The time point used by other groups to identify *Loxosceles* venom in the blood of rabbits or patients might have been too long (after 1 day) to detect venom. Barbaro and co-workers investigated the presence of human antibodies against the venom of treated patients, instead of the venom itself. In this work, a small number of samples (20%) were positive for the presence of antibodies, but these molecules could be found even 67 days after the bite [31]. The authors raised the hypothesis that patients who received immunotherapy could have a suppressor effect for antibody production or even sequestration of antibody complexes from circulation.

For spiders from the genus *Phoneutria*, *Atrax* and *Hadronyche* attempts to identify venoms were made exclusively using serum as the biological sample. This could be due to the kinetics of these venoms, which act more systemically than locally, in contrast to *Loxosceles* venoms. Conversely, the amount of venom injected by these genera is probably greater, based on the average amount of milked venom for these spiders (in the range of mg) [32,33]. In the case of *Phoneutria* venom, analogous to the *Loxosceles* work [30], *Phoneutria* venom was found after up to 8 h in sera of experimentally envenomed mice [34]. *Phoneutria* venom was also detected in one human blood sample of a systemic envenomation case in the range of ng/mL (Table 3) [33,35]. Remarkably, Miller and co-workers [33] were able to find *Atrax* and *Hadronyche* venom in samples stored at −80 °C up to 10 years after collection. Furthermore, this research group tested a greater number of human samples (9 samples) and could detect venom in 6 of them, which could lead to more reliable results (Table 3), considering the sparse number of reported cases from these spiders every year. 

Altogether, these studies show that distinct types of biological samples must be investigated considering different spider genera. *Loxosceles* spider venom acts more locally; therefore, most of the venom can be found close to the bite site (such as hair, skin exudate and biopsy), and venom is generally undetectable in serum by techniques at the tested time-points. Conversely, for funnel-web and armed spiders, there is no information available about the amount of venom that could be recovered at the injection site. However, all studies were able to identify venom in the serum of the majority of envenomated patients (although the number of patients tested was very low). 

The sandwich format of the enzyme-linked immunosorbent assay (ELISA), using antibodies against the whole venom, was the method of choice in most works. This could be due to the widespread use of this technique in diagnostic assays. Moreover, most of the studies discussed in this review have a low number of animals (3 animals at most), and additionally, human samples are generally the product of a unique clinical case, when considering spider accidents. Negative controls were usually material extracted from a contralateral region of the patient, and never from samples of the same population with no envenomation. Furthermore, information obtained with these data must consider that the kinetics of spider envenomation pathogenesis in animal models is very different from that in humans (necrosis following *Loxosceles* bites appears 12 h after envenomation in rabbits and days after envenomation in humans) [26]. Nevertheless, the knowledge acquired from these studies can serve as the basis for the development of a new diagnostic assay.

### 2.2. Scorpions 

Despite the importance that scorpion venom identification and quantification in patients represents for scorpionism treatment, few studies reported so far have addressed this issue. As most scorpion stings are usually very painful, patients report the accident more accurately to clinicians. However, the identification of scorpion species and quantification of circulating venom in victims remains an important matter for better treatment intervention.

Chavez-Olórtegui and colleagues successfully detected *Tityus serrulatus* venom in the sera of hospitalized patients with systemic envenomation, but failed to distinguish mild cases (*n* = 27; 67.5% of patients in the study) from controls (*n* = 15) (Table 2) [36]. In this test, when mild cases were excluded, sensitivity was 94.7%, but when all cases of scorpion sting were included, sensitivity decreased to 39.3% [18]. Nonetheless, the assay was useful to confirm that envenoming severity in patients stung by *T. serrulatus* is related to plasma venom concentrations [18].

Similarly, other studies have aimed to detect and quantify scorpion venom antigens in the sera of envenomed patients (e.g., *Androctonus australis garzonii*, *Buthus occitanus tunetanus* [21], *Androctonus mauretanicus mauretanicus*, *Buthus occitanus* [37], *Tityus discrepans* [38] and *Centruroides limpidus limpidus* [39]). In some of these studies, ELISA assays were more accurately calibrated and some technical aspects were improved, e.g., the elimination of cross-reacting and heterophilic antibodies [21]. As a result, they were able to distinguish mildly envenomed patients from the control group and to better assess envenoming severity. Furthermore, most of these studies showed that patients with moderate and severe envenoming grades had a higher venom concentration [39] in sera over a longer period of time [37,38]. 

Additionally, it was demonstrated that ELISA, using specific antibodies for a given scorpion species, can be used for scorpion venom quantification within other species from the same genus [36,40] and even from different genera [37], due to venom antigen similarity. For example, Chase and colleagues [40] evaluated the developed ELISA using antibodies against Mexican *Centruroides* sp. for determining venom serum levels after envenomation by *Centruroides sculpturatus* from the United States. The assay was also tested using venom from another *Centruroides* species (*Centruroides limpidus limpidus*), and the standard curves were very similar, providing evidence of highly analogous venoms among *Centruroides* species. Cross-reactivity in ELISA was also demonstrated using antibodies produced against *A. mauretanicus mauretanicus* with the venom of the scorpion *Buthus occitanus* [37]. 

In addition to ELISA, a radioactive method was used to quantify *Androctonus australis garzonii* venom in the serum of experimentally envenomed rabbits. It was found that scorpion toxin plasma concentrations determined by radioactivity were significantly higher than those determined by ELISA. This could be due to degradation/cleavage of toxins that might occur in vivo, and the fact that degraded toxins could still be quantified by radioactivity but not by ELISA [41] (Table 4).

One study used a very different approach, Reverse Passive Arthus (RPA) reaction, to discriminate between Buthidae and Scorpionidae scorpion envenomation in mice. The test detected *Mesobuthus* venom, presenting 84.44% sensitivity and 100% of specificity. According to the authors, the simplicity of application, low cost, minimal risk, suitable specificity, sensitivity and quick visible results indicated the possibility of using this reaction in envenomation diagnosis. However, despite satisfactory results, more research is required to prove and reduce probable side effects before it is tested in humans [42].

Altogether, we can observe that the main technology used for identification and quantification of scorpion venom was sandwich ELISA using polyclonal antibodies, similar to spider detection studies. Most studies used the toxic fraction of scorpion venom to obtain specific antibodies for ELISA and evaluated venom concentration in the sera of hospitalized scorpion-stung patients. Only one study evaluated venom concentration in urine using ELISA, although in only one patient [40]. Sera of individuals from the same population that had not been stung by scorpions were generally used as controls. It is noteworthy that the sample size regarding venom detection in scorpion-stung patients is considerably larger than in studies involving spider victims, due to the higher prevalence of scorpionism. Having such a prominent data set enabled some studies to discriminate even between controls, mild, moderate and severe accidents, at statistically significant levels [21,43]. Other studies have also shown a correlation between envenoming grade and venom levels, but failed to discriminate controls from mild cases [19,36,39]. However, they are still relevant to helping clinicians in therapeutic decisions for a victim’s treatment. These observations stress the importance of conducting large trials to validate diagnostic tests. 

## 3. In Vitro Assays for the Establishment of Antivenom Effectiveness

### 3.1. Spiders 

Spider venom detection in biological samples can go beyond diagnostic purposes. Some studies have employed immunological detection of venom components to confirm antivenom efficacy in removing circulating venom in arachnidism victims, by comparing venom concentrations before and after antivenom treatment (Table 3). 

Brazilian anti-arachnidic antivenom, produced by equine immunization with a venom mixture from spiders *Loxosceles gaucho* and *Phoneutria nigriventer* and from scorpions of the genus *Tityus*, had its efficacy accessed experimentally and clinically. Toro and colleagues, in 2006, used venom detection by sandwich ELISA to demonstrate the ability of anti-arachnidic antivenom in neutralizing the toxic activities of three venoms, and its capacity to remove venom from circulation in experimentally envenomed mice. The assay did not detect circulating venom in the first 15 min after treatment for the three venoms tested, indicating the efficacy of the anti-arachnidic antivenom [44].

The conditions used in this study are not comparable to what happens in real human accidents, but a subsequent work validated this antivenom efficacy, at least for *P. nigriventer* envenomation. In a case reported by Bucaretchi et al. in 2008, the adult victim analyzed sought medical assistance 4 h after the accident. At this time point, it was possible to detect 47.5 ng/mL of venom in the patient’s blood. After antivenom treatment with 5 vials of anti-arachnidic antivenom, venom could no longer be detected, and systemic manifestations in the envenomed patient disappeared 1 h later [35].

Australian funnel-web spider envenomation is also primarily treated with antivenom. Aiming to improve its use, Miller et al. measured both venom and antivenom concentrations in 9 samples of envenomed patients, with a specifically developed sandwich ELISA, using biotinylated antivenom for detection [33]. In three atypical mild cases, venom was not detected, suggesting the possibility that they were caused by other spider species, or they were a result of dry bites or the assay was not sensitive enough to detect such a small amount of venom. In the six additional cases, a level of 0.4 to 35 ng/mL of venom was detected, and this amount decreased to nearly zero after specific antivenom administration, showing the efficacy of the antivenom in removing venom from circulation. This work concluded that no more than 2 vials of antivenom were needed to treat envenomation and, although treatments with up to 17 vials have been reported [45], they appear to be unnecessary. Despite efficient venom removal, some envenoming effects could not be reversed, indicating that perhaps venom components that are already attached to their targets cannot be unbound or that once a secondary host response is triggered, the provoked envenomation effect cannot be reversed, regardless of the excess of antivenom [33]. 

### 3.2. Scorpions 

As observed in the studies for spiders, despite the development of improved antivenom preparations for scorpions, antivenom treatment efficacy is still controversial, particularly in cases of mild and moderate envenomation. In addition, guidelines for antivenom immunotherapy have not been rigorously determined, and the use of such specific treatment is still justified only based on empirical observations [20,46]. Thus, most authors have used ELISA technique in experimental and/or clinical studies to determine scorpion venom pharmacokinetics and to evaluate the effectiveness of serotherapy for the treatment of scorpion envenomation.

It has been shown that the anti-scorpionic antivenom produced by Fundação Ezequiel Dias (FUNED), administered intravenously, was efficient in neutralizing circulating *T. serrulatus* venom antigens, reducing envenomation symptoms in mice [47] and in hospitalized patients [48]. In the experiments conducted in mice, significant venom reduction could be observed in serum, as well as in different organs (lungs, heart, liver, spleen, kidney), as soon as 15 min after treatment. However, although antivenom (applied 1 h after envenoming) could achieve complete clearance of circulating venom, trace venom amounts were still detected in organs [47]. The polyvalent anti-arachnidic antivenom produced by BUTANTAN Institute was also efficient in removing *T. serrulatus* venom from circulation in mouse models [44]. As the first study [47] does not mention the concentration of the antivenom used, it is not possible to compare the effectiveness of the two antivenoms or even to estimate the optimum effective antivenom therapeutic dosage, because none of them was tested at different dosages. Research addressing these issues is still lacking. Moreover, recent data have shown that anti-scorpionic and anti-arachnidic antivenoms (both from BUTANTAN) failed to neutralize toxic enzymatic activities from distinct species of *Tityus* venom in vitro [49], pointing once more to the need for better achievement of antivenom efficacy.

A series of studies concerning antivenom efficacy against scorpions from *Androctonus* and *Buthus* genus in North African countries has been conducted over the years. It was shown that the application of bivalent anti-*A.australis garzoni* and anti-*B.occitanus tunetanus* antivenom was not efficient in reducing venom plasma concentrations when administered in a single dose by the intramuscular route. However, when a second intramuscular antivenom injection (IM) was administered, total venom clearance was achieved after 6 h [21,43]. When antivenom was administered intravenously (IV), a single dose reduced venom concentration to less than 1 ng/mL, 63 min after treatment. A second intravenous antivenom injection eliminated circulating venom in 30 min, regardless of the administration route of the first injection (IM or IV). This work stresses the importance of a correct therapeutic protocol to achieve better treatment performance. 

The efficacy of anti-*A. mauretanicus mauretanicus* antivenom was assessed in 275 patients envenomed with either *Androctonus* or *Buthus* venoms in Morocco. The results showed that patients that received 10 mL of antivenom had better clinical recovery and lower circulating venom levels than patients who received a 2–5 mL dosage or who did not receive any antivenom [37]. Although authors reported that different routes of antivenom injection were used in the analyzed patients (intramuscular in 77.6%, subcutaneous in 6.2% and both routes in 16.2% of cases), the possible outcome differences for each approach were not discussed, despite the fact that complete venom clearance was not achieved for any of the protocols used in this work. The intravenous route of antivenom administration was not explored, making it impossible to compare the results of this study with the previous ones reported here. 

To better understand the importance of the administration route as well as the influence of time for antivenom injection and dosage, Krifi and colleagues confirmed in rabbits the superiority of the intravenous route over the intramuscular one and also reported the requirement of a minimum antivenom dose to achieve full circulating venom neutralization [46]. They also attested to the fact that delayed antivenom immunotherapy is still beneficial, as it can significantly reduce the remaining circulating venom. These findings were also corroborated by a study that evaluated the efficacy of serotherapy against *Androctonus australis* venom in Algeria [20]. The results indicated that envenomed patients treated with one vial of antivenom, administered intramuscularly, did not show a significant decrease in the amounts of circulating venom, suggesting that antivenom efficacy must be evaluated using different antivenoms doses and different routes of administration.

Confirming that delayed antivenom immunotherapy is still efficient in neutralizing remaining plasma toxins in experimental envenomated animals, a study analyzing toxicokinetic parameters of *A. australis garzonii* toxic fraction in experimentally envenomed rabbits showed that immunotherapy performed 5 h after venom injection immediately decreased venom toxin levels in plasma. This work also showed superior performance of intravenous antivenom injection [41]. In this study, two different venom detection techniques were used: ELISA and radioactivity of 125I-labeled toxic fraction Aag-FG50 after trichloroacetic acid (TCA) precipitation. The curves produced for both methods differed in venom pharmacokinetic analysis, with radioactivity counting apparently being capable of still detecting venom even when complete clearance appeared to have been achieved by ELISA. These differences could be explained by a partial degradation of the radiolabeled toxins and/or by an absorption of radioactivity to precipitable plasma proteins, but neither hypothesis was confirmed. 

Regarding the analysis of antivenom efficacy against Mexican scorpion species, Osnaya-Romero and colleagues also confirmed by ELISA the efficacy of the commercial polyspecific F(ab’)2 anti-scorpion antivenom Alacramyn^®^ in reducing serum venom levels and envenoming symptoms in children stung by *Centruroides limpidus limpidus* [39]. As soon as 30 min after immunotherapy, the amount of toxin detected in the serum of patients decreased to almost undetectable levels, and this reduction correlated with the improvement of clinical conditions. 

In summary, all studies reported here support the benefits of antivenom administration to patients with systemic manifestations of scorpion envenoming, as they demonstrate reduction of circulating venom upon antivenom administration. They indicated that serotherapy is particularly effective when antivenom is administered as soon as possible after venom injection. Nevertheless, even in cases of treatment delay, intravenous antivenom should still be prescribed, because of venom’s slow elimination half-life [41,46,50]. The intravenous route seems to have superior efficiency when compared to intramuscular antivenom administration. Using an adequate antivenom dosage is also important for completely neutralizing circulating venom, avoiding the resurgence of venom concentrations that were not fully bound to antivenom and minimizing allergic reaction due to large loads of protein injection. However, none of the studies presented here attempted to determine exactly what this dosage should be, considering a correlation with the detected venom levels in the admission of patients. Thus, well-designed studies are still needed to define the optimal dosage and time for application of antivenom, based on the venom serum concentration in each patient. To achieve this goal, better venom detection assays in biological samples are still needed.

## 4. Biotechnological Limitations and Perspectives for Arachnid Venom Detection 

**Figure 1:**
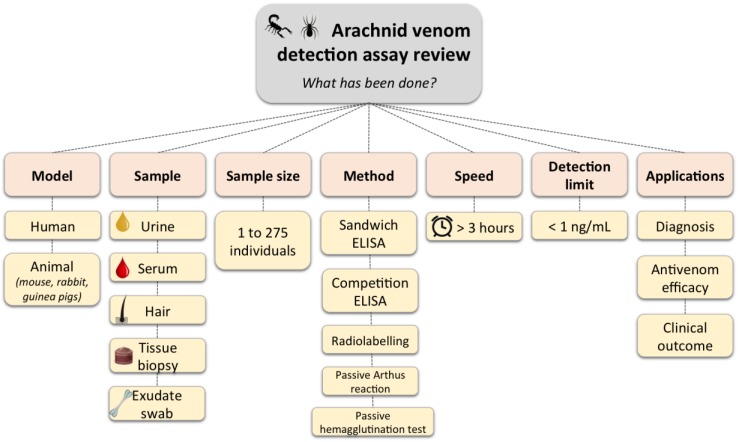
Summary of existing arachnid venom detection assay variables.

It is important to identify and quantify arachnid venoms, and there is a lack of standardization and clinical tests to address this issue. Therefore, this section will discuss the biological performance of past attempts at arachnid venom detection, such as the nature of biological samples, the experimental model, and sample size, as well as technical limitations, such as the speed and sensitivity of the tests (Figure 1).

Considering the biological samples used to identify and quantify venoms, some diversity can be observed in the reviewed papers, at least for spiders. Although tissue biopsy and hair have been used as samples with success in some studies, especially in loxoscelism, they are not samples commonly used in ordinary health facilities. Lesion swabs, urine and saliva are less invasive options to be considered; still, it has been demonstrated that most venoms are not particularly concentrated in these samples, which would require tests with very low detection limits. Some studies on spider diagnosis and almost all studies considering scorpionism have used serum as the sample to be assayed. The only report of scorpion venom quantification in urine showed very similar venom concentrations in urine and serum, but was conducted in only one patient. Despite the fact that venom elimination in animals occurs mainly through urine [51], no data are available for humans. Although more invasive, using blood/serum as the sample of choice for envenoming diagnosis would not require many changes in patient management, since blood collection is part of the routine of health facilities. 

Most studies employed the sandwich ELISA technique to probe venoms in biological samples, since the presence of two antibodies can increase specificity, giving results that are more reliable. Due to its simplicity, low-cost reagents, and capability of being read in batches, ELISA seems a valuable technique for diagnosis in the low-resource contexts in which envenomation usually takes place.

Both antibodies used in the ELISA tests proposed (capture and detection) were invariably polyclonal within the articles. Polyclonal antibodies are an undefined mixture of molecules that can suffer variations from batch to batch and are more likely to produce undesirable cross-reactivity. To minimize these possible unspecific reactions, in some cases reported in this review, polyclonal antibodies were pre-incubated with sera from false positive samples placed in an affinity column, eliminating the presence of heterophilic antibodies [20,21]. This additional step seemed to increase the quality of the results. 

The production of monoclonal antibodies, which would increase specificity and purity, could be an alternative. Although hybridoma production is a long, expensive and labor-intensive technique [52], some work has been done aiming toward anti-arachnid toxin monoclonal antibody production [53,54,55,56,57,58,59,60]. Aptamers can also be an alternative sensor for venom detection, as already described for snake toxins [61,62], and for sphingomyelinase D from the *Loxosceles laeta* spider [63]. Despite these achievements, further experimental validation for diagnostic purposes using these mAbs and aptamers, particularly employing biological samples, has not been explored thoroughly yet. Better sensors must be explored, since it has been shown that the most significant contributor to the detection limit of an immunoassay is the affinity between the binding pairs [64].

Besides the nature of the sample, the method of choice, and the kind of sensor used for venom detection, the time consumed in the test is one limiting step. From sample preparation to result analysis, time should be very short to minimize venom damage and to assist treatment approach decisions. The typical ELISA technique employed for a venom search requires at least 3–4 h to retrieve results. Faster assay methods, such as point-of-care methodologies [65] or studies attempting to optimize incubation periods in already existing tests, as well as the improvement of result analysis through standardization, should be pursued to better suit the needs of management of envenomed patients.

Another critical step for arachnid diagnostic assays is the limit of detection, considering that, although very toxic, arachnid venom is injected in much reduced amounts in victims. The average amount of venom milked from a *Loxosceles* spider is a few tenths of a microliter, containing 30–50 μg of venom [26,66]. On average, 1.25 mg is milked from a male *Athrax* spider [33]. Assuming that the quantity injected during a human envenomation is the amount retrieved by milking, and taking into consideration that an adult male weighing 70 kg has a blood volume of about 5 L, the concentration found in the serum (if all the venom reaches this body compartment at the same time) is, at maximum, in the low range of ng/mL in the case of *Loxosceles* envenomation. 

Most papers investigated herein, and the majority of commercial colorimetric ELISA kits, have detection limits on the order of ng/mL for protein analytes, which would be suitable for detecting envenomings occurring only under the above-reported optimum conditions. Mild and moderate envenoming victims may have circulating venom concentrations that fall below this detection limit. Considering physiological venom clearance and venom binding to target organs, venom concentrations should decrease even more over time. Although some manuscripts in this review claim to have performed tests with detection limits lower than pg/mL, if the sample chosen for identifying venom is blood, tests with a better limit of detection should be applied to contemplate the diagnosis of these less severe accidents, which are the most difficult ones to identify properly. To enhance the detection signal, and therefore the limit of detection, streptavidin and biotin detection strategies have been used by some groups described in this review [22,27,38]. Another strategy applied with satisfactory results is the use of ELISA in the competition format [29], as well as radioactive methods to evaluate venom kinetics [41]. However, until now, no work has attempted to achieve more sensitive signals with more refined techniques, such as radioimmunoassays or chemiluminescent, fluorescent and dynamic light scattering systems, perhaps due to the need for sophisticated laboratory equipment [67,68]. In the case of radioimmunoassay, another hindering factor could be the handling of radioactive isotopes, which requires appropriate facilities and special licenses [69]

To increase sensitivity and specificity, tests based on nucleic-acid amplification technology, like PCR, could also be an alternative, as it has already been pursued for snake envenomation [70]. Immuno-PCR (iPCR), which was first developed by Sano et al. in 1992 [71], could be an alternative approach to increase the detection limit in envenomation diagnosis but has not been explored yet for the diagnosis of envenomed patients.

There are other up-to-date methods currently used in diagnosis of other diseases that could be applied in envenomings. Gene expression alterations have been identified in patients suffering from different infectious and inflammatory diseases [72,73,74,75,76], and can also be detected as a result of envenomation [77,78,79]. Using high-throughput methodologies, a transcriptomic signature of these specific gene expression alterations (either from mRNA or miRNA) can be defined in the victim’s biological samples and used as a biomarker for developing diagnostic tools. Another approach using chemical signatures instead of RNAs [80,81] can also be applied, using mass spectrometry (MS) for detecting small-molecule metabolites or other biomarkers produced by envenomings [82]. Establishing and validating molecular profiles or biomarker cartography for envenomations can be challenging, and the fast dynamics of the onset of envenoming symptoms can hamper the clinical relevance of this type of test. Due to its sensitivity, it is possible that mass spectrometry can also detect and quantify venom components directly [83], but, to the best of our knowledge, this has not been tested on biological samples of envenomed patients yet. Mass spectrometry can also be combined with immunoassays by using immunoaffinity separation of the samples before MS detection, which can be advantageous when using complex biological samples [84]. Although it is evident that these innovative techniques may produce diagnostic tests with more specific and sensitive results, there is the problem of cost, expertise and equipment requirements, which may not be accessible to most healthcare facilities in developing countries, where most envenoming accidents take place [85].

Looking at the whole picture of scorpion and spiders diagnostic assay development, we have noticed a knowledge gap. Efforts to improve arachnid envenomation diagnostic assay, validated in animals or humans (mandatory to belong to this review), were made especially in the late 1990s and early 2000s. After this date, very few papers concerning this matter were released, and none of the proposed tests were followed up. It is worth mentioning that the number of accidents has not decreased in this period, highlighting the urgency of the development of methods to improve the healthcare of envenomed victims. 

This lack of studies can be explained by other factors. Some of the reasons for this window in time could be the establishment of ethical approval requirements, which are slow to accomplish, especially in developing countries (the appearance of ethical committees took place in 1990) and the holding back of the indiscriminate use of animals [86]. Another hypothesis is that the ELISA technique, which is one of the most widely used in vitro diagnostic methods for detection and protein analysis [87] was already well explored in these initial papers and, since then, no new low-cost and sensitive methods have emerged for this purpose. In addition, the vast majority of accidents involving arachnids occurs in developing countries, where the budget to be invested in R&D is limited, making it an unattractive market for diagnostic companies to invest, thus causing its slow evolution. 

After reviewing what has been done over the years in terms of arachnid venom detection assays, it was possible to detect some gaps and no clinical application of the proposed tests for envenomation diagnosis. Different samples and different assay formats have been proposed, but only a few studies follow a well-designed study to validate the test, which is essential to translate it into actual applications in clinics. Therefore, we propose some ideas to build upon this knowledge, to better design validation assays leading to the approval and application of arachnid envenoming diagnosis.

## 5. Unmet Needs for Arachnid Diagnosis and Recommendations to Validate an Efficient Test

Diagnosis is a very important healthcare asset for successful implementation of treatment for various conditions such as infectious diseases, cancer, and neurological disorders, as well as for prevention and implementation of health policies. For these aforementioned medical conditions, there are several diagnostic techniques, as well as clinical trials and evaluation studies attesting to their efficacy [88]. 

In the case of envenoming diagnosis, especially concerning arachnids, we can observe from this review that the information available is very preliminary, compared to what has been done in other fields. In Table 5, we suggest some unmet needs in diagnosis for venomous arachnid accidents, and below, we further discuss some important points to be considered to achieve this goal.

As shown in Table 5, an in vitro diagnostic test for arachnid envenomation can be pursued for different needs and objectives, such as identification of the accident itself (when the bite/sting is not witnessed or is painless); to identify animal genera or species (when misjudged by patients and clinicians), leading to different treatment approaches; to quantify venom levels and correlate with clinical outcomes; to evaluate antivenom treatment effectiveness; to establish the appropriate number of antivenom vials needed for effective treatment; or even to obtain epidemiological data to enhance government healthcare policies. 

**Figure 2:**
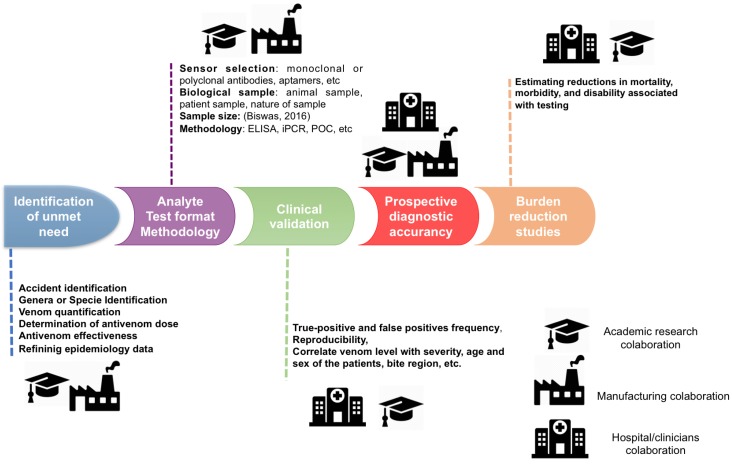
Five-phase roadmap for clinical validation of the arachnid diagnostic test.

A diagnostic test can only be applied efficiently once it is validated analytically and clinically. A validation study should be carefully planned to avoid bias and to assess the real benefits and flaws of the evaluated test [89]. Based on a proposition by Frisoni et al. in 2017 [90], and adapted from a cancer biomarker study [91], we suggest a five-phase road-map for the development and clinical validation of arachnid diagnostic tests (Figure 2). For arachnid envenomation, one can consider Phase 1 as the definition of the problem to be solved (listed in Table 5). Phase 2 evaluates the analytical aspects, in which pre-clinical exploratory studies should define the test format, methodology and analytes. Phase 3 concerns clinical viability aspects such as the frequency of true positives and false positives of the assay in tested patients, reproducibility and correlation with variables such as levels of venom detected with clinical severity, age and sex of the patients. In phase 4, prospective diagnostic accuracy in a clinical setting is contemplated, and phase 5 relates to mortality and morbidity rate estimation. In the papers reviewed herein, phase 2 has barely been achieved in some of the studies, while the other phases have never been reached.

The analytical aspects that should be addressed in diagnosis validation studies include specificity, sensitivity, reproducibility, accuracy and limits of detection. Among the reviewed papers, the sample size used in the analytical phase, specificity, and sensitivity were poorly defined and reported. The number of samples analyzed fluctuates from one clinical case to a few hundred for the same genera of animals responsible for envenomation (see Table 1, Table 2, Table 3 and Table 4). It is key to estimate sample size, which can be estimated as suggested in [89]. It should be based on the prevalence of the envenomation and on what degree of sensitivity (or specificity) is expected. 

Both retrospective and prospective evaluations should be considered for analytical and clinical validation. As shown by Miller and co-workers [33], retrospective studies for analytical validation could be an alternative for species which have a sparse number of cases a year, yet still have an important clinical outcome. Although retrospective assessment is convenient, fast and economical, the available patients’ information could be limited and patients’ consent for the test should be asked in advance [92]. Concerning patients’ consent, ethical committee approval and eligibility criteria, little information can be found in most of the manuscripts reviewed in this article. As has already been discussed, this could be due to the late implementation of ethical committees in developing countries. 

Within the samples collected, reference gold standards should be rationalized, since there are no approved tests or controls for arachnid envenomation to date. Patients who bring the animal responsible for the accident, for instance, can be considered to be ‘gold standard’ positive control samples to validate methodologies. Likewise, an algorithm based on clinical symptoms should be designed.

Another issue that should be considered in diagnosis development and validation is cost-effectiveness and potential clinical impact. If diagnostic results do not have an impact on the detected condition management, be it clinical handling or epidemiology, at a feasible cost, one should question its utility [93,94]. This evaluation should contemplate cost saving in resource utilization due to proper diagnosis (such as antivenom amount used to treat victims), as well as effects on morbidity/mortality rates and time elapsed for hospital discharge of rapidly diagnosed patients compared to non-diagnosed ones [95]. The benefits of patient outcome are rarely reported properly but should be addressed in test validation studies [96]. Van den Bruel and colleagues [97] proposed to include these features in diagnostic tests validation studies, in addition to technical, clinical aspects and test accuracy.

After design and validation, proper implementation of a diagnostic test in a clinical setting is a decisive step that converts all the research work done to the benefit to a patient’s clinical outcome or towards improving epidemiology management. A given methodology should be appropriate for the conditions under which it will be applied, considering the physical and technological structure. End-user education and training are also important, because it is not sufficient to have a rapid test if the results are not obtained in time to influence medical conduct [95]. 

In summary, this review addresses the efforts described in the literature to diagnose arachnid envenomation, presenting the gaps in the field and proposing strategies to overcome these gaps. Arachnid venom detection in bitten/stung patients affects the correct identification of the species responsible for the accident, and consequently the correct treatment intervention of these patients. Furthermore, the detection of venom in a patient’s sample is crucial to determining the efficacy of the antivenom treatment. To obtain an effective diagnostic test, it is essential for between health centers, clinicians, researchers and industry to join forces for the development of sensitive sensors and assays, the collection of quality samples, and proper test validations. 

## 6. Materials and Methods

The search for diagnostic studies of arachnid envenomation or antivenom effectiveness was done by a systematic interrogation of Ovid MEDLINE^®^ search using the Ovid interface (http://www.ovid.com/site/platforms/ovidsp.jsp) with different combinations of the words “detection” or “diagnose*” or “recognize” or “screening” or “assessment” or “evaluation” with the names of medically relevant arachnid genera (“Loxosceles” or “Phoneutria” or “Latrodectus” or “Atrax” or “Hadronyche”, for spiders and “Leiurus” or “Androctonus” or “Buthus” or “Mesobuthus” or “Parabuthus” or “Tityus” or “Centruroides”) for scorpions. The last searches were done in December 2017. The bibliographical search was further pursued by following the references of selected articles to find other publications. All retrieved manuscripts were manually inspected. Only articles in English, expressly mentioning the goal of diagnosis (or venom detection and quantification or antivenom detection) validated using samples from envenomated animals or humans in title or abstract were retained for this study. Additionally, only papers regarding venomous arachnids were selected in this review. We discarded articles which did not make explicit reference to identification or quantification of venom or antivenom, and those that did not have full-text available or provided by the authors. Likewise, papers without validation using animals or human samples and not taking into account medically important arachnid species were excluded.

## Figures and Tables

**Table 1:** In vitro assays for identification and/or quantification of spider venoms.

Target	Sample	Model	Technology	Amount Injected/Route	Time after Injection	Amount Detected	Detection Limit	Reference
IgG anti-*Loxosceles* venom	Serum	Human (*n* = 20)	Indirect ELISA	Bite	9 to 120 days	Detectable in 4 patients	ND	[31]
*Loxosceles* venom	Skin exudate	Guinea pigs (*n*=26)	Passive Hemagglutination Inhibition Test	24 μg/i.d	Up to 3 days	Detectable	ND	[23]
	Serum	Mouse (*n* = 10)	Sandwich ELISA	2.5 μg/s.c	0.25 h	Not detectable	<0.1 ng	[30]
					0.50 h	60 ng/mL		
					1.0 h	45 ng/mL		
					2.0 h	40 ng/mL		
					4.0 h	10 ng/mL		
					8.0 h	5 ng/mL		
					12.0 h, 1, 2 and 3 days	Not detectable		
	Biopsy	Human (*n* = 1)	Competitive ELISA	Bite	4 days	3350 pg/2-mm biopsy	ND	[29]
	Hair		Competitive ELISA (Strep-Biot)			1113 pg/100 μL		
	Biopsy	Rabbits (*n* = 3)	Sandwich ELISA	3 μg/i.d	1 day	4280 pg/4-mm biopsy	<0.1 ng	[27]
	Biopsy	Rabbits (*n* = 3)	Sandwich ELISA	3 μg/i.d	7 days	205 pg/4-mm biopsy	<0.1 ng	[28]
	Serum				1, 2, 3, 4 and 7 days	Not detectable		
	Hair				1 day	216 pg/100 μL		
					2 days	192 pg/100 μL		
					3 days	172 pg/100 μL		
					4 days	148 pg/100 μL		
					7 days	80 pg/100 μL		
*Loxosceles* venom	Skin exudate	Human (*n* = 1)	Sandwich ELISA	Bite	3 days	34 pg/100 μL	24 pg	[24]
	Serum					Not detectable		
	Skin exudate	Human (*n* = 1)	Sandwich ELISA	Bite	13 days	Detectable	<0.1 ng	[25]
	Skin exudate	Rabbits (*n* = 3)	Sandwich ELISA	4–5 μg/s.c	7, 10, 14 and 21 days	~5 pg	ND	[22]
	Biopsy				1 and 3 days	Not detectable		
*Phoneutria* venom	Serum	Mice (*n* = 5)	Sandwich ELISA	5 μg/s.c	0.50 h	25 ng/mL	<2 ng	[34]
		Human (*n* = 2)		Bite	ND	11–26 ng/mL		

Abbreviations: i.d: intradermic; s.c: subcutaneous; ND: not determined; Strep-Biot: streptavidin-biotin.

**Table 2:** In vitro assays for identification and/or quantification of scorpion venoms.

Target	Sample	Model	Technology	Amount Injected/Route	Time after Injection	Amount Detected *(Normalized)*	Detection Limit	Reference
*Tityus* venom	Serum	Human (*n* = 40)	Sandwich ELISA	Sting	ND	*	0.1 ng/mL	[36]
	Serum	Mice (*n* = 10)	Sandwich ELISA	1 µg/s.c	0.5 h	*	0.1 ng/mL	
	Serum	Human (*n* = 56)	Sandwich ELISA	Sting	ND	*	4.8 ng/mL	[19]
	Serum	Human (*n* = 19)	Sandwich ELISA	Sting	1.5 h	2.14–50 ng/mL	ND	[18]
	Serum	Human (*n* = 205)	Sandwich ELISA (Strep-Biot)	Sting	0.5–6.0 h	0.09–202 ng/mL	0.09 ng/mL	[38]
*Androctonus* and *Buthus* venom	Serum	Human (*n* = 180)	Sandwich ELISA	Sting	5 to 4.8 h	GI-0.9 to 4.2 ng/mL	0.9 ng/mL	[21]
						GII-3 to 16 ng/mL		
						GIII-13 to 38 ng/mL		
*Centruroides* venom	Serum	Human (*n* = 3)	Sandwich ELISA	Sting	50 min–5.2 h	8.2–29.7 ng/mL	1 ng/mL	[40]
	Urine				~490 min–8.2 h	9.0 ng/mL		
*Mesobuthus* venom	Skin exudate	Mice (*n* = 6)	Reverse passive Arthus reaction (RPA)	100 μg/s.c	45 min	Detectable in ≈ 84.4%	ND	[42]

Abbreviations: s.c: subcutaneous; ND: not determined; Strep-Biot: streptavidin-biotin; GI: grade I envenomation (mild); GII: grade II envenomation (moderate); GIII: grade III envenomation (severe); *: could not be determined in reference article.

**Table 3:** Spider antivenom effectiveness measured in serum by sandwich ELISA.

		Antigen			Antivenom					
Target	Model	Amount Injected/Route	Time after Injection	Amount Detected	Antivenom	Amount Injected/Route	Time after Venom for Antivenom Injection	Amount Detected (Time after Antivenom Injection)	Detection Limit	Reference
*Loxosceles* venom	Mice (*n* = 4)	10 µg i.d	0.25 h	20 ng/mL	Anti-arachnidic (BUTANTAN)	0.2 mL/animal i.v	Immediately after venom	Not detectable in all time points (0.25 h, 0.5 h, 1.0 h, 4.0 h and 24 h)	2 ng/mL	[44]
			0.5 h	28 ng/mL						
			1.0 h	18 ng/mL						
			4.0 h	7 ng/mL						
			24.0 h	0 ng/mL						
*Phoneutria* venom	Mice (*n* = 4)	10 µg i.d	0.25 h	65 ng/mL	Anti-arachnidic (BUTANTAN)	0.2 mL/animal i.v	Immediately after venom	1 ng/mL (0.25 h)	2 ng/mL	[44]
			0.5 h	50 ng/mL				1 ng/mL (0.5 h)		
			1.0 h	48 ng/mL				1 ng/mL (1.0 h)		
			4.0 h	17 ng/mL				Not detectable (4.0 h)		
			24.0 h	0 ng/mL				Not detectable (24 h)		
	Human (*n* = 1)	Bite	4.0 h	47.5 ng/mL	Anti-arachnidic (BUTANTAN)	5 vials i.v	4.0 h	Not detectable in all time points (1.0 h, 6.0 h, 24 h and 48 h)	17.1 ng/mL	[35]
*Atrax* and *Hadronyche* venom **	Human (*n* = 9)	Bite	ND	0.4–35 ng/mL	Anti-funnel-web spider (CSL)	2–12 vials i.v	1.0–5.0 h	Not detectable to 1 ng/mL (various time points)	0.2 ng/mL	[33]

**Abbreviations:** i.d: intradermic; s.c: subcutaneous; ND: not determined; **: streptavidin-biotin ELISA used; BUTANTAN: Butantan Institute, São Paulo, Brazil; CSL: Commonwealth Serum Laboratories, Melbourne, Australia.

**Table 4:** Scorpion antivenom effectiveness measured in serum.

Antigen					Antivenom					
Target	Model	Amount Injected/Route	Time after Injection	Amount Detected	Antivenom	Amount Injected/Route	Time after Venom for Antivenom Injection	Amount Detected (Time after Antivenom Injection)	Detection Limit	Reference
*Tityus* venom	Mice (*n* = 28)	10 μg/s.c	0.25 h	760 ng/mL	Anti-scorpionic (FUNED)	10 μL/i.v	Immediately after venom	41 ng/mL (0.25 h)	ND	[47]
			0.5 h	780 ng/mL				50 ng/mL (0.5 h)		
			1.0 h	400 ng/mL				100 ng/mL (1 h)		
			2.0 h	360 ng/mL				100 ng/mL (2 h)		
			4.0 h	50 ng/mL				40 ng/mL (4 h)		
			8.0 h	0 ng/mL				Not detectable (8 h)		
							1.0 h	Not detectable in all time points		
	Human (*n* = 18)	Sting	ND	≈15.07 ng/mL	Anti-scorpionic (FUNED)	5–30 mL/i.v	ND	Not detectable (1.0 h)	0.1 ng/mL	[48]
	Mice (*n* = 4)	10 µg/i.d	0.25 h	34 ng/mL	Anti-arachnidic (BUTANTAN)	0.2 mL/animal i.v	Immediately after venom	Not detectable in all time points	2 ng/mL	[44]
			0.5 h	25 ng/mL						
			1.0 h	28 ng/mL						
			4.0 h	18 ng/mL						
			24.0 h	0 ng/mL						
*Androctonus* venom	Human (*n* = 40)	Sting	Up to 2.0 h	GI (*n* = 31): 0.96 ± 0.36 ng/mL	Horse F(ab′)2 anti-AahFG50	10 mL/i.m	ND	0.63 ± 0.19 ng/mL (1.0 h)	0.5 ng/mL	[20]
								0.49 ± 0.15 ng/mL (3.0 h)		
				GII (*n* = 9): 2.44 ± 1.1 ng/mL				1.11 ± 0.57 ng/mL (1.0 h)		
								0.63 ± 0.21 ng/mL (3.0 h)		
	Rabbit (*n* = 3) ^#^	100 μg/kg **** s.c	0–13.0 h	***	Anti-BotFG50 purified from Bivalent anti-Aag and anti-Bot (PIT)	80, 8, 4 or 0.8 mg/i.m or i.v	1.0 h	***	ND	[41]
*Androctonus* and *Buthus* venom	Human (*n* = 147)	Sting	ND	≈23 ng/mL (*n* = 12)	Bivalent anti-Aag and anti-Bot (PIT)	No antivenom		6 ± 1 ng/mL (6.0 h)	0.9 ng/mL	[43]
				≈24 ng/mL (*n* = 28)		1 × 2 mL/kg (10–30 mL)/i.m	At hospital admission	4 ± 2 ng/mL (6.0 h)		
				*** (*n* = 34)		1 × 2 mL/kg (10–30 mL)/i.v		3 ± 1 ng/mL (1.0 h)		
				≈13 ng/mL (*n* = 16)		2 × 2 mL /kg (5–15 mL)/i.m	1st at hospital admission 2nd 1 h later	Not detectable (6.0 h after 2nd injection)		
				*** (*n* = 42)		2 × 2 mL/kg (5–15 mL)/i.m/i.v		Not detectable (0.5 h after 2nd injection)		
				*** (*n* = 15)		2 × 2 mL /kg (5–15 mL)/i.v		Not detectable (0.5 h after 2nd injection)		
	Human (*n* = 275)	Sting	5 min–16.0 h (1.27 ± 1.6 h)	≈18.72 ng/mL (*n* = 96)	Horse anti-*A.mauretanicus mauretanicus*	No antivenom		22.20 ± 27.04 ng/mL (3.0 h)	0.78 ng/mL	[37]
				≈12.10 ng/mL (*n* = 48)		2–5 mL		9.86 ± 14.86 ng/mL (1.0 h)		
							ND	8.81 ± 15.63 ng/mL (3.0 h)		
				≈19.35 ng/mL (*n* = 131)		10 mL		6.64 ± 11.12 ng/mL (1.0 h)		
								4.98 ± 15.25 ng/mL (3.0 h)		
*Buthus* venom	Rabbit (*n* = 3–6)	100 μg/kg s.c	5 min–4 8h	***	Anti-BotFG50 purified from Bivalent anti-Aag and anti-Bot (PIT)	30, 12 and 6 mg/kg in 1 mL i.m or i.v	0.5–3.0 h	***	ND	[46]
*Centruroides* venom	Human (*n* = 44)	Sting	ND	0–87.4 ng/mL	Alacramyn^®^ (Bioclon, Mexico)	ND	ND	Not detectable to 4.7 ng/mL (0.5 h)	0.1 ng/mL	[39]

Abbreviations: i.d: intradermic; i.m: intramuscular; i.v: intravenous; s.c: subcutaneous; ND: not determined; PIT: Pasteur Institute of Tunis; ^#^: tested also by radiolabeling; GI: grade I envenomation (mild); GII: grade II envenomation (moderate; GIII: grade III envenomation (severe); ***: only toxokinetic curves available in reference article, with no determined individual values; ****: containing a trace amount of radiolabeled 125I-venom for radioactivity detection.

**Table 5:** Unmet needs for arachnid diagnosis.

Genera	Arachnid Genera Identification from Venom Detected in Human Biological Samples	Diagnostic Test for Clinical Use	Venom Quantification or/and Correlation with Patient Clinical Outcome	Determination of Antivenom Dose for Human Treatment	Determination of Antivenom Treatment Effectiveness in Patients	Refining of Epidemiology Data	Sample Size and References
*Loxosceles*	**✔✔**	**✖**	**✖**	**✖**	**✖**	**✖**	*n* = 30 [30]; *n* = 1 [24]
*Latrodectus*	**✖**	**✖**	**✖**	**✖**	**✖**	**✖**	
*Phoneutria*	**✔**	**✖**	**✔**	**✖**	**✖**	**✖**	*n* = 2 [34]; *n* = 1 [35]
*Atrax*/*Hadronyche*	**✔**	**✖**	**✔**	**✔**	**✔**	**✖**	*n* = 9 [33]
*Leiurus*	**✖**	**✖**	**✖**	**✖**	**✖**	**✖**	
*Androctonus*	**✔✔✔**	**✖**	**✔✔✔**	**✔✔✔**	**✔✔✔**	**✖**	*n* = 180 [21]; *n* = 147 [43]; *n* = 275 [37]
*Buthus*	**✔✔✔**	**✖**	**✔✔✔**	**✔✔✔**	**✔✔✔**	**✖**	*n* =180 [21]; *n* = 147 [43]; *n* = 275 [37]
*Tityus*	**✔✔✔**	**✖**	**✔✔✔**	**✖**	**✔✔**	**✖**	*n* = 205 [38]; *n* = 18 [51] *n* = 40 [36]; *n* = 56 [19]; *n* = 19 [18]
*Centruroides*	**✔✔**	**✖**	**✔✔**	**✖**	**✔✔**	**✖**	*n* = 3 [40]; *n* = 44 [39]
*Mesobuthus*	**✖**	**✖**	**✖**	**✖**	**✖**	**✖**	
*Parabuthus*	**✖**	**✖**	**✖**	**✖**	**✖**	**✖**	

Abbreviations: ****✖**** not accomplished; **✔**accomplished with sample size 1–10; **✔✔** accomplished with sample size 11–50; **✔✔✔** accomplished with sample size >50.

## References

[1] Cordeiro F.A., Amorim F.G., Anjolette F.A.P., Arantes E.C. (2015). Arachnids of medical importance in Brazil: Main active compounds present in scorpion and spider venoms and tick saliva. J. Venom. Anim. Toxins Incl. Trop. Dis..

[2] Junghanss T., Bodio M. (2006). Medically Important Venomous Animals: Biology, Prevention, First Aid, and Clinical Management. Clin. Infect. Dis..

[3] Garb J.E., González A., Gillespie R.G. (2004). The black widow spider genus Latrodectus (Araneae: Theridiidae): Phylogeny, biogeography, and invasion history. Mol. Phylogenet. Evol..

[4] Gremski L.H., Trevisan-Silva D., Ferrer V.P., Matsubara F.H., Meissner G.O., Wille A.C.M., Vuitika L., Dias-Lopes C., Ullah A., de Moraes F.R. (2014). Recent advances in the understanding of brown spider venoms: From the biology of spiders to the molecular mechanisms of toxins. Toxicon.

[5] Vetter R.S., Isbister G.K. (2008). Medical aspects of spider bites. Annu. Rev. Entomol..

[6] Isbister G.K., Fan H.W. (2011). Spider bite. Lancet.

[7] Lucas S.M. (2015). The history of venomous spider identification, venom extraction methods and antivenom production: A long journey at the Butantan Institute, São Paulo, Brazil. J. Venom. Anim. Toxins Incl. Trop. Dis..

[8] Bawaskar H.S., Bawaskar P.H. (2012). Scorpion sting. J. Assoc. Phys. India.

[9] Isbister G.K., Bawaskar H.S. (2014). Scorpion Envenomation. N. Engl. J. Med..

[10] Boyer L.V., Theodorou A.A., Berg R.A., Mallie J., Chávez-Méndez A., García-Ubbelohde W., Hardiman S., Alagón A. (2009). Antivenom for Critically Ill Children with Neurotoxicity from Scorpion Stings. N. Engl. J. Med..

[11] Chippaux J.-P. (2012). Emerging options for the management of scorpion stings. Drug Des. Dev. Ther..

[12] Gaver-Wainwright M.M., Zack R.S., Foradori M.J., Lavine L.C. (2011). Misdiagnosis of Spider Bites: Bacterial Associates, Mechanical Pathogen Transfer, and Hemolytic Potential of Venom From the Hobo Spider, Tegenaria agrestis (Araneae: Agelenidae). J. Med. Entomol..

[13] Mowry J.B., Spyker D.A., Cantilena L.R., McMillan N., Ford M. (2014). 2013 Annual Report of the American Association of Poison Control Centers’ National Poison Data System (NPDS): 31st Annual Report. Clin. Toxicol..

[14] Bennett R.G., Vetter R.S. (2004). An approach to spider bites. Erroneous attribution of dermonecrotic lesions to brown recluse or hobo spider bites in Canada. Can. Fam. Phys..

[15] Vetter R.S. (2009). Arachnids misidentified as brown recluse spiders by medical personnel and other authorities in North America. Toxicon.

[16] Vetter R.S., Cushing P.E., Crawford R.L., Royce L.A. (2003). Diagnoses of brown recluse spider bites (loxoscelism) greatly outnumber actual verifications of the spider in four western American states. Toxicon.

[17] Swanson D.L., Vetter R.S. (2005). Bites of brown recluse spiders and suspected necrotic arachnidism. N. Engl. J. Med..

[18] Rezende N.A.D.E., Chavez-Olortegui C., Amaral C.F.S. (1996). Is the severity of Tityus serrulatus scorpion envenoming related to plasma venom concentrations?. Toxicon.

[19] Rezende N.A., de Dias M.B., Campolina D., Chavez-Olortegui C., Amaral C.F.S. (1995). Standardization of an enzyme linked immunosorbent assay (ELISA) for detecting circulating toxic venom antigens in patients stung by the scorpion Tityus serrulatus. Rev. Inst. Med. Trop. São Paulo.

[20] Hammoudi-triki D., Ferquel E., Robbe-vincent A., Bon C., Choumet V., Laraba-djebari F. (2004). Epidemiological data, clinical admission gradation and biological quantification by ELISA of scorpion envenomations in Algeria: Effect of immunotherapy. Trans. R. Soc. Trop. Med. Hyg..

[21] Krifi M.N., Kharrat H., Zghal K., Abdouli M., Abroug F., Bouchoucha S., Dellagi K., El Ayeb M. (1998). Development of an ELISA for the detection of scorpion venoms in sera of humans envenomed by Androctonus australis garzonii (AAG) and Buthus occitanus tunetanus (BOT): Correlation with clinical severity of envenoming in Tunisia. Toxicon.

[22] McGlasson D.L., Green J.A., Stoecker W.V., Babcock J.L., Calcara D.A. (2009). Duration of Loxosceles reclusa venom detection by ELISA from swabs. Clin. Lab. Sci..

[23] Barrett S.M., Romine-Jenkins M., Blick K.E. (1993). Passive hemagglutination inhibition test for diagnosis of brown recluse spider bite envenomation. Clin. Chem..

[24] Stoecker W.V., Green J.A., Gomez H.F. (2006). Diagnosis of loxoscelism in a child confirmed with an enzyme-linked immunosorbent assay and noninvasive tissue sampling. J. Am. Acad. Dermatol..

[25] Keklikci U., Akdeniz S., Sakalar Y.B., Cakmak S.S., Unlu K. (2008). Loxosceles reclusa bite to the eyelid. Eur. J. Ophthalmol..

[26] Pauli I., Minozzo J.C., Henrique D.A., Silva P., Chaim O.M., Veiga S.S. (2009). Analysis of therapeutic benefits of antivenin at different time intervals after experimental envenomation in rabbits by venom of the brown spider (*Loxosceles intermedia*). Toxicon.

[27] Gomez H.F., Greenfield D.M., Miller M.J., Warren J.S. (2001). Direct correlation between diffusion of Loxosceles reclusa venom and extent of dermal inflammation. Acad. Emerg. Med..

[28] Krywko D.M., Gomez H.F. (2002). Detection of Loxosceles species venom in dermal lesions: A comparison of 4 venom recovery methods. Ann. Emerg. Med..

[29] Miller M.J., Gomez H.F., Snider R.J., Stephens E.L., Czop R.M., Warren J.S. (2000). Detection of Loxosceles venom in lesional hair shafts and skin: Application of a specific immunoassay to identify dermonecrotic arachnidism. Am. J. Emerg. Med..

[30] Chávez-Olórtegui C., Zanetti V.C., Ferreira A.P., Minozzo J.C., Mangili O.C., Gubert I.C. (1998). ELISA for the detection of venom antigens in experimental and clinical envenoming by Loxosceles intermedia spiders. Toxicon.

[31] Barbaro K.C., Cardoso J.L.C., Eickstedt V.R.D., Mota I. (1992). IgG antibodies to *Loxosceles* sp. spider venom in human envenoming. Toxicon.

[32] Lucas S. (1988). Spiders in Brazil. Toxicon.

[33] Miller M., Leary M.A.O., Isbister G.K. (2016). Towards rationalisation of antivenom use in funnel-web spider envenoming: Enzyme immunoassays for venom concentrations. Clin. Toxicol..

[34] Chávez-Olórtegui C., Bohórquez K., Alvarenga L.M., Kalapothakis E., Campolina D., Maria W.S., Diniz C.R. (2001). Sandwich-ELISA detection of venom antigens in envenoming by *Phoneutria nigriventer* spider. Toxicon.

[35] Bucaretchi F., Mello S.M., Vieira R.J., Mamoni R.L., Blotta M.H., Antunes E., Hyslop S. (2008). Systemic envenomation caused by the wandering spider *Phoneutria nigriventer*, with quantification of circulating venom. Clin. Toxicol..

[36] Chávez-Olórtegui C., Fonseca S.C., Campolina D., Amaral C.F., Diniz C.R. (1994). ELISA for the detection of toxic antigens in experimental and clinical envenoming by *Tityus serrulatus* scorpion venom. Toxicon.

[37] Ghalim N., El-hafny B., Sebti F., Heikel J., Lazar N., Moustanir R., Benslimane A. (2000). Scorpion envenomation and serotherapy in Morocco. Am. J. Trop. Med..

[38] D’Suze G., Moncada S., Gonzales C., Sevcik C., Aguilar V., Alagon A. (2003). Relationship between plasmatic levels of various cytokines, tumour necrosis factor, enzymes, glucose and venom concentration following Tityus scorpion sting. Toxicon.

[39] Osnaya-Romero N., Acosta-Saavedra L.C., Goytia-Acevedo R., Lares-Asseff I., Basurto-Celaya G., Perez-Guille G., Possani L.D., Calderón-Aranda E.S. (2016). Serum level of scorpion toxins, electrolytes and electrocardiogram alterations in Mexican children envenomed by scorpion sting. Toxicon.

[40] Chase P., Boyer-Hassen L., Mcnally J., Vazquez H.L., Theodorou A.A., Walter F.G., Alagon A. (2009). Serum levels and urine detection of *Centruroides sculpturatus* venom in significantly envenomated patients. Clin. Toxicol..

[41] Krifi M.N., Savin S., Debray M., Bon C., El Ayeb M., Choumet V. (2005). Pharmacokinetic studies of scorpion venom before and after antivenom immunotherapy. Toxicon.

[42] Khoobdel M., Nikbakhtboroujeni G., Zahraeisalehi T., Khosravi M., Sasani F., Bokaei S., Koochakzadeh A., Zamani-Ahmadmahmudi M., Akbari A. (2014). Diagnosis of Mesobuthus eupeus envenomation by skin test: Reverse passive Arthus reaction. Toxicon.

[43] Krifi M.N., Amri F., Kharrat H., El Ayeb M. (1999). Evaluation of antivenom therapy in children severely envenomed by *Androctonus australis* garzonii (Aag) and Buthus occitanus tunetanus (Bot) scorpions. Toxicon.

[44] Toro A.F., Malta M.B., Soares S.L., Rocha G.C., da Lira M., De Oliveira T.A., Takehara H.A., Lopes-Ferreira M., Santoro M.L., Guidolin R. (2006). Role of IgG (T) and IgGa isotypes obtained from arachnidic antivenom to neutralize toxic activities of Loxosceles gaucho, Phoneutria nigriventer and Tityus serrulatus venoms. Toxicon.

[45] Isbister G.K., Gray M.R., Balit C.R., Raven R.J., Stokes B.J., Porges K., Tankel A.S., Turner E., White J., Fisher M.M. (2005). Funnel-web spider bite: A Systematic Review of Recorded Clinical Cases. Med. J. Aust..

[46] Krifi M.N., Miled K., Abderrazek M., El Ayeb M. (2001). Effects of antivenom on Buthus occitanus tunetanus (Bot) scorpion venom pharmacokinetics: Towards an optimization of antivenom immunotherapy in a rabbit model. Toxicon.

[47] Revelo M.P., Bambirra E.A., Ferreira A.P., Diniz C.R., Chavez-Olortegui C. (1996). Body distribution of Tityus serrulatus scorpion venom in mice and effects of scorpion antivenom. Toxicon.

[48] Rezende N.A., Amaral C.F., Freire-maia L. (1998). Immunotherapy for scorpion envenoming in Brazil. Toxicon.

[49] Venancio E.J., Portaro F.C.V., Kuniyoshi A.K., Carvalho D.C., Pidde-Queiroz G., Tambourgi D.V. (2013). Enzymatic properties of venoms from Brazilian scorpions of Tityus genus and the neutralisation potential of therapeutical antivenoms. Toxicon.

[50] Santana G.C., Freire A.C.T., Ferreira A.P.L., Chavez-Olortegui C., Diniz C.R., Freire-Maia L. (1996). Pharmacokinetics of Tityus serrulatus scorpion venom determined by enzyme-linked immunosorbent assay in the rat. Toxicon.

[51] Ismail M. (1995). The scorpion envenomation syndrome. Toxicon.

[52] Michnick S.W., Sidhu S.S. (2008). Submitting antibodies to binding arbitration. Nat. Chem. Biol..

[53] Alvarenga L.M., Avila R.A.M., De Amim P.R., Martins M.S. (2005). Molecular characterization of a neutralizing murine monoclonal antibody against Tityus serrulatus scorpion venom. Toxicon.

[54] Alvarenga L.M., Martins M.S., Moura J.F., Oliveira C., Mangili O.C., Granier C., Chávez-Olórtegui C. (2003). Production of monoclonal antibodies capable of neutralizing dermonecrotic activity of *Loxosceles* intermedia spider venom and their use in a specific immunometric assay. Toxicon.

[55] Clot-faybesse O., Juin M., Roche H., Devaux C. (1999). Monoclonal antibodies against the Androctonus australis hector scorpion neurotoxin I: Characterisation and use for venom neutralisation. FEBS Lett..

[56] Devaux C., Clot-faybesse O., Juin M., Mabrouk K., Sabatier J.-M., Rochat H. (1997). Monoclonal antibodies neutralizing the toxin II from Androctonus australis hector scorpion venom: Usefulness of a synthetic, non-toxic analog. FEBS Lett..

[57] Dias-Lopes C., Felicori L., Rubrecht L., Cobo S., Molina L., Nguyen C., Galéa P., Granier C., Molina F., Chávez-Olortegui C. (2014). Generation and molecular characterization of a monoclonal antibody reactive with conserved epitope in sphingomyelinases D from Loxosceles spider venoms. Vaccine.

[58] Guilherme P., Fernandes I., Barbaro K.C. (2001). Neutralization of dermonecrotic and lethal activities and differences among 32–35 kDa toxins of medically important Loxosceles spider venoms in Brazil revealed by monoclonal antibodies. Toxicon.

[59] Jiacomini I., Silva S.K., Aubrey N., Muzard J., Chavez-olortegui C., Moura J., De Moura J., Billiald P., Alvarenga L.M. (2016). Immunodetection of the “brown” spider (*Loxosceles* intermedia) dermonecrotoxin with an scFv-alkaline phosphatase fusion protein. Immunol. Lett..

[60] Pucca M.B., Zoccal K.F., Roncolato E.C., Bertolini T.B., Campos L.B., Cologna C.T., Faccioli L.H., Arantes E.C., Barbosa J.E. (2012). Serrumab: A human monoclonal antibody that counters the biochemical and immunological effects of Tityus serrulatus venom. J. Immunotoxicol..

[61] Lauridsen L.H., Shamaileh H.A., Edwards S.L., Taran E., Veedu R.N. (2012). Rapid one-step selection method for generating nucleic acid aptamers: Development of a DNA Aptamer against α-bungarotoxin. PLoS ONE.

[62] Ye F., Zheng Y., Wang X., Tan X., Zhang T., Xin W., Wang J., Huang Y., Fan Q., Wang J. (2014). Recognition of Bungarus multicinctus Venom by a DNA Aptamer against β-Bungarotoxin. PLoS ONE.

[63] Sapag A., Salinas-luypaert C., Constenla-muñoz C. (2014). First report of in vitro selection of RNA aptamers targeted to recombinant *Loxosceles* laeta spider toxins. Biol. Res..

[64] Zhang S., D’Angeli A.G., Brennan J.P., Huo Q. (2014). Predicting detection limits of enzyme-linked immunosorbent assay (ELISA) and bioanalytical techniques in general. Analyst.

[65] Drain P.K., Hyle E.P., Noubary F., Freedberg K.A., Wilson D., Bishai W.R., Rodriguez W., Bassett I.V. (2014). Diagnostic point-of-care tests in resource-limited settings. Lancet Infect. Dis..

[66] Tambourgi D.V., De F., Fernandes Pedrosa M., van den Berg C.W., Goncalves-de-Andrade R.M., Ferracini M., Paixao-Cavalcante D., Morgan B.P., Rushmere N.K. (2004). Molecular cloning, expression, function and immunoreactivities of members of a gene family of sphingomyelinases from Loxosceles venom glands. Mol. Immunol..

[67] Tighe P.J., Ryder R.R., Todd I., Fairclough L.C. (2015). ELISA in the multiplex era: Potentials and pitfalls. Proteom.-Clin. Appl..

[68] Vial S., Wenger J. (2017). Single-step homogeneous immunoassay for detecting prostate-specific antigen using dual-color light scattering of metal nanoparticles. Analyst.

[69] Dominguez J.A., Matas L., Manterola J.M., Blavia R., Sopena N., Belda F.J., Padilla E., Giménez M., Sabrià M., Morera J. (1997). Comparison of Radioimmunoassay and Enzyme Immunoassay Kits for Detection of Legionella pneumophila Serogroup 1 Antigen in Both Concentrated and Nonconcentrated Urine Samples. J. Clin. Microbiol..

[70] Suntrarachun S., Pakmanee N., Tirawatnapong T., Chanhome L., Sitprija V. (2001). Development of a polymerase chain reaction to distinguish monocellate cobra (Naja khouthia) bites from other common Thai snake species, using both venom extracts and bite-site swabs. Toxicon.

[71] Sano T., Smith C., Cantor C. (1992). Immuno-PCR: Very sensitive antigen detection by means of specific antibody-DNA conjugates. Science.

[72] Fehlbaum-Beurdeley P., Sol O., Désiré L., Touchon J., Dantoine T., Vercelletto M., Gabelle A., Jarrige A.C., Haddad R., Lemarié J.C. (2012). Validation of AclarusDx^TM^, a Blood-Based Transcriptomic Signature for the Diagnosis of Alzheimer’s Disease. J. Alzheimers Dis..

[73] Zadel M., Maver A., Kovanda A., Peterlin B. (2018). Transcriptomic Biomarkers for Huntington’ s Disease: Are gene expression signatures in whole blood reliable biomarkers?. OMICS.

[74] Darboe F., Mbandi S.K., Thompson E.G., Fisher M., Rodo M., Rooyen M., van Rooyen M., Filander E., Bilek N., Mabwe S. (2018). Diagnostic performance of an optimized transcriptomic signature of risk of tuberculosis in cryopreserved peripheral blood mononuclear cells. Tuberculosis.

[75] Bochukova E.G., Lawler K., Croizier S., Keogh J.M., Patel N., Strohbehn G., Lo K.K., Humphrey J., Hokken-Koelega A., Damen L. (2018). A Transcriptomic Signature of the Hypothalamic Response to Fasting and BDNF Deficiency in Prader-Willi Syndrome. Cell Rep..

[76] Nikolayeva I., Bost P., Casademont I., Duong V., Koeth F., Prot M., Czerwinska U., Ly S., Bleakley K., Cantaert T. (2018). A blood RNA signature detecting severe disease in young dengue patients at hospital arrival. J. Infect. Dis..

[77] Corzo G., Espino-Solis G.P. (2017). Selected scorpion toxin exposures induce cytokine release in human peripheral blood mononuclear cells. Toxicon.

[78] Gehrie E.A., Nian H., Young P.P. (2013). Brown Recluse spider bite mediated hemolysis: Clinical features, a possible role for complement inhibitor therapy, and reduced RBC surface glycophorin A as a potential biomarker of venom exposure. PLoS ONE.

[79] Dantas R.T., Sampaio T.L., Lima D.B., Menezes B., Canuto J.A., Toyama M.H., Evangelista M., Martins C. (2018). Evaluation of KIM-1 as an early biomarker of snakebite-induced AKI in mice. Toxicon.

[80] McCall L., Morton J.T., Bernatchez J.A., Jair L., Knight R., Dorrestein P.C., McKerrow J.H. (2017). Mass Spectrometry-Based Chemical Cartography of a Cardiac Parasitic Infection. Anal. Chem..

[81] Cho Y., Su H., Wu W., Wu D., Hou M., Kuo C., Shiea J. (2015). Biomarker Characterization by MALDI–TOF/MS. Adv. Clin. Chem..

[82] Pham T.V., Piersma S.R., Oudgenoeg G., Jimenez C.R. (2012). Label-free mass spectrometry- based proteomics for biomarker discovery and validation. Expert Rev..

[83] Calderón-Celis F., Cid-Barrio L., Ruiz Encinar J., Sanz-Medel A., Calvete J.J. (2017). Absolute venomics: Absolute quantification of intact venom proteins through elemental mass spectrometry. J. Proteom..

[84] Nedelkov D. (2012). Mass spectrometry-based protein assays for in vitro diagnostic testing. Expert Rev..

[85] Mabey D., Peeling R.W., Ustianowski A., Perkins M.D. (2004). Diagnostics for the developing world. Nat. Rev. Microbiol..

[86] Chippaux J.-P. (2015). Epidemiology of envenomations by terrestrial venomous animals in Brazil based on case reporting: From obvious facts to contingencies. J. Venom. Anim. Toxins Incl. Trop. Dis..

[87] Gan S.D., Patel K.R. (2013). Enzyme immunoassay and enzyme-linked immunosorbent assay. J. Inve. Dermatol..

[88] Bossuyt P.M., Reitsma J.B., Bruns D.E., Gatsonis C.A., Glasziou P.P., Irwig L., Lijmer J.G., Moher D., Rennie D., De Vet H.C. (2015). STARD 2015: An updated list of essential items for reporting diagnostic accuracy studies. Clin. Chem..

[89] Biswas B. (2016). Clinical Performance Evaluation of Molecular Diagnostic Tests. J. Mol. Diagn..

[90] Frisoni G.B., Boccardi M., Barkhof F., Blennow K., Cappa S., Chiotis K., Démonet J.F., Garibotto V., Giannakopoulos P., Gietl A. (2017). Strategic roadmap for an early diagnosis of Alzheimer’s disease based on biomarkers. Lancet Neurol..

[91] Pepe M.S., Etzioni R., Feng Z., Potter J.D., Thompson M.L., Thornquist M., Winget M., Yasui Y. (2001). Phases of Biomarker Development for Early Detection of Cancer. J. Natl. Cancer Inst..

[92] Banoo S., Bell D., Bossuyt P., Herring A., Mabey D., Poole F., Smith P.G., Sriram N., Wongsrichanalai C., Linke R. (2010). Evaluation of diagnostic tests for infectious diseases: General principles. Nat. Rev. Microbiol..

[93] Bossuyt P.M.M., Reitsma J.B., Linnet K., Moons K.G.M. (2012). Beyond Diagnostic Accuracy: The Clinical Utility of Diagnostic Tests. Clin. Chem..

[94] Ferrante di Ruffano L., Hyde C.J., Mccaffery K.J., Bossuyt P.M.M., Deeks J.J. (2012). Assessing the value of diagnostic tests: A framework for designing and evaluating trials. BMJ.

[95] Messacar K., Parker S.K., Todd J.K., Dominguez S.R. (2017). Implementation of Rapid Molecular Infectious Disease Diagnostics: The Role of Diagnostic and Antimicrobial Stewardship. J. Clin. Microbiol..

[96] Siontis K.C., Siontis G.C.M., Contopoulos-ioannidis D.G., Ioannidis J.P.A. (2014). Diagnostic tests often fail to lead to changes in patient outcomes. J. Clin. Epidemiol..

[97] Van den Bruel A., Cleemput I., Aertgeerts B., Ramaekers D., Buntinx F. (2007). The evaluation of diagnostic tests: Evidence on technical and diagnostic accuracy, impact on patient outcome and cost-effectiveness is needed. J. Clin. Epidemiol..

